# Incidence and clinical characteristics of multiple myeloma with low M-protein levels and normal values of hemoglobin, creatinine, calcium, and serum free light chain ratio

**DOI:** 10.1038/s41408-021-00460-0

**Published:** 2021-04-07

**Authors:** Agoston Gyula Szabo, Tobias Wirenfeldt Klausen, Niels Abildgaard, Henrik Gregersen, Trine Silkjær, Per Trøllund Pedersen, Robert Schou Pedersen, Carsten Helleberg, Emil Hermansen, Brian Iversen Schnack, Annette Juul Vangsted

**Affiliations:** 1 Department of Hematology Vejle Hospital, Vejle, Denmark; 2 Department of Hematology Herlev University Hospital, Herlev, Denmark; 3 Department of Hematology Odense University Hospital, Odense, Denmark; 4 Department of Hematology Aalborg University Hospital, Aalborg, Denmark; 5 Department of Hematology Aarhus University Hospital, Aarhus, Denmark; 6 Department of Hematology Esbjerg Hospital, Esbjerg, Denmark; 7 Department of Hematology Regionshospitalet Holstebro, Holstebro, Denmark; 8 Department of Hematology Zealand University Hospital, Roskilde, Denmark; 9 Department of Hematology Rigshospitalet, Copenhagen, Denmark

**Keywords:** Myeloma, Signs and symptoms

Dear Editor,

The presence of an M-protein in the serum is a common incidental finding. It can be associated with infections, inflammatory conditions and autoimmune diseases, and the asymptomatic precursor conditions monoclonal gammopathy of undetermined significance (MGUS) and smoldering multiple myeloma (SMM). Only one percent of individuals with MGUS progress to a malignant plasma cell or lymphoproliferative disease per year^1,2^ and an important predictor of progression is the level of the M-protein at the time of diagnosis, which is used in the risk-stratification model by the Mayo Clinic^2–4^. Early diagnosis and treatment of multiple myeloma (MM) is important, as delays may result in life-threatening infections, painful bone fractures, or dialysis-dependent renal failure^5–9^. Moreover, there is increasing evidence supporting treatment of patients with SMM and this question is being explored in several ongoing clinical trials^10^. The serum free light chain (sFLC) assay, imaging of the skeleton, a bone marrow biopsy, and blood tests are part of the diagnostic work-up for MM, but if clinical evaluation and blood tests suggest low risk MGUS, a baseline bone marrow examination and skeletal radiography are not recommended by the International Myeloma Working Group^3,11^.

In a recently published retrospective review from the Mayo Clinic, Sidiqi et al. reported that 29 (1.3%) of 2225 MM patients had laboratory values within reference range for calcium, creatinine, hemoglobin, a sFLC ratio <100, and absence of lytic lesions assessed by conventional skeletal survey. These patients would have been misclassified as SMM or MGUS without bone marrow biopsy or advanced imaging^12^.

The aim of our study was to describe the incidence of MM and SMM with an MGUS-like profile (defined as MM with IgG M-protein ≤ 1.5 g/dl or an IgA M-protein ≤ 1.0 g/dl and with normal levels of ionized calcium, creatinine, and hemoglobin) since 2005 in Denmark. Furthermore, we wanted to characterize the patients with MGUS-like MM who also had a normal sFLC ratio at diagnosis. Finally, we wanted to understand the reasons for referral for diagnostic work-up for these MM patients with no apparent signs of organ damage.

We used the population-based Danish Multiple Myeloma Registry (DMMR), which includes clinical data for all patients with MM and SMM diagnosed since 2005^13–15^. Measurements of sFLC have been recorded in the DMMR since 2012. A sFLC ratio of 0.26–1.65 at diagnosis was defined as normal. Patients with LDH levels above normal were standardized according to age. In patients with age >70 years the upper limit was ≥255 UL, and in patients with age ≤70 the upper limit was ≥205 UL. Hypogammaglobulinemia was defined qualitatively as one or more of uninvolved immunoglobulins below normal levels (IgG < 6.1 g/L, IgA < 0.70 g/L, IgM < 0.39 g/L). High-risk cytogenetics were defined as the presence of del17p, t(4;14), or t(14;16) with a cut-off of 10%. Patients with AL amyloidosis, medullary compression syndrome, peripheral neuropathy, or dialysis-dependent renal failure at presentation were excluded. The incidence of MGUS-like MM patients was age-adjusted to the EU 2013 population. Causes of diagnostic work-up were reviewed by audit of medical records. Data were entered in a Research Electronic Data Capture (REDcap) database and merged with the baseline characteristics from DMMR.

At the time of data cut-off for this study (3rd April 2019), the DMMR included 5116 newly diagnosed myeloma patients (Fig. 1A). We found a significant increase in the age-adjusted incidence of MM between 2005 and 2017 (*p* < 0.001; Fig. 1B). This increase was also present in MGUS-like MM with an IgG M-protein ≤1.5 g/dl or an IgA M-protein ≤1.0 g/dl with normal hemoglobin, creatinine, and ionized calcium at diagnosis (*p* < 0.0001; Fig. 1B). The increase in the incidence of MM correlated significantly with a wider use of sensitive imaging techniques in the diagnostic work-up, such as CT, PET-CT, or MRI (*r* = 0.86; *p* = 0.0002; Supplementary Fig. 1). The diagnostic sFLC ratio had a stronger correlation to the bone marrow clonal plasma cell percentage compared to the diagnostic M-protein concentrations (Supplementary Figs. 2 and 3).

**Fig. 1:**
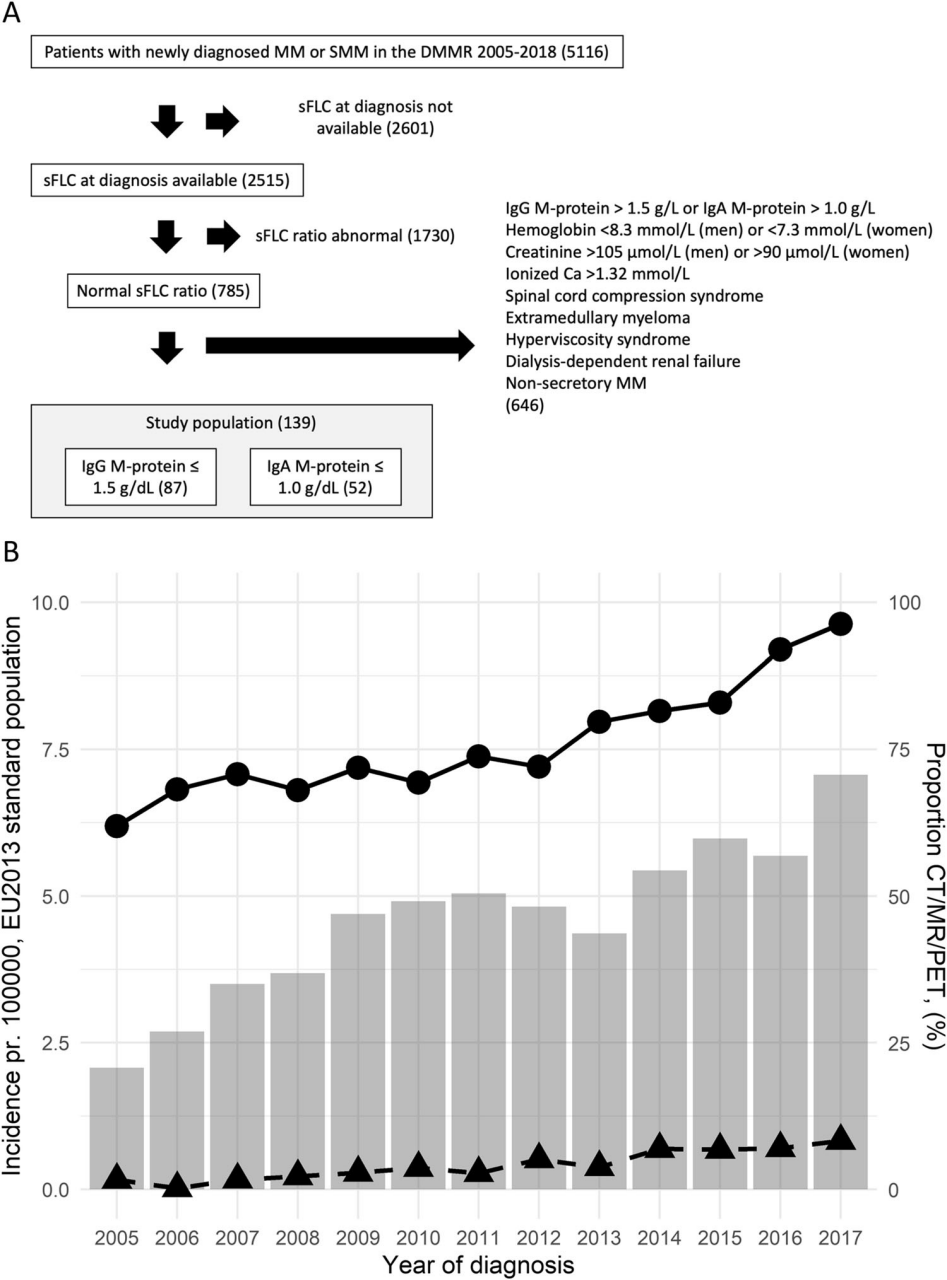
Flow-chart for study inclusion and the incidence of multiple myeloma in Denmark. **A** Flow-chart for study inclusion: Among 5116 patients diagnosed with MM or SMM and registered in the DMMR, a diagnostic sFLC result was available in 2515 patients and 785 had a normal sFLC ratio. Of these patients, 139 had an MGUS-like phenotype IgG M-protein ≤1.5 g/dl or an IgA M-protein ≤1.0 g/dl and with normal levels of normal hemoglobin, creatinine, ionized calcium and no amyloidosis, medullary compression syndrome, extramedullary myeloma, peripheral neuropathy or dialysis-dependent renal failure at presentation. MM multiple myeloma, SMM smoldering myeloma, sFLC serum free light chain. **B** Incidence of multiple myeloma in Denmark: The columns with values displayed on the left vertical axis show the incidence of multiple myeloma pr. 100.000 in Denmark, age adjusted to the EU 2013 standard population. Filled circles present the age-adjusted incidence for all MM patients and the filled triangles present the age-adjusted incidence of myeloma patients with an IgG M-protein ≤1.5 g/dL and IgA M-protein ≤1.0 g/dL and normal blood levels of ionized calcium, creatinine, and hemoglobin. The gray bars with values displayed on the right vertical axis show the percentage of patients assessed with sensitive imaging techniques like CT, MRI, and PET (CT/MR/PET) as part of the diagnostic work-up. The years of diagnosis in the calendar period 2005–2017 are shown on the horizontal axis. Patients diagnosed in 2018 were not fully registered at data cut-off.

We identified 139 patients (5.5% of patients in the DMMR with available sFLC results), with MGUS-like MM. The clinical characteristics of these patients are presented in Supplementary Table 1. Osteolytic lesions were found in 23.9%. No patients had higher than 60% bone marrow clonal plasma cell infiltration. The results of FISH analysis were available in 51.1% of the patients. High-risk cytogenetics were found in 12.7% of patients. Hypogammaglobulinemia, elevated Beta-2-microglobulin and elevated LDH were found in 52.9%, 34.5%, and 15.4% of patients, respectively.

Causes of diagnostic work-up were available in all patients but one (Supplementary Table 2). Eighty (58.0%) patients were referred with symptoms, most frequently bone pain and fatigue. Ninety-one (65.9%) patients were referred with abnormal blood tests, most frequently the presence of a serum M-protein. Fourteen (10.1%) patients were referred with an abnormal skeletal imaging result. Eight (5.8%) patients were referred because of a biopsy finding. The number of patients without bone pain or abnormal skeletal imaging results at referral was 92 (67.2%), of these 38 (41.8%) had bone lesions. Fifty-eight patients (42.0%) did not have any symptoms at referral; of these 27 (29.5%) had bone lesions.

Data from the DMMR show an increase in the age-adjusted incidence of MM over the years that correlates with the wider use of sensitive imaging techniques. The incidence of MGUS-like MM has also increased from 0.12/100.000 in the calendar period 2005–2007 to 0.74/100.000 in the calendar period 2015–2017. In daily practice, the decision whether a bone marrow biopsy and sensitive imaging techniques should be performed in such patients is difficult, and there is an inherent risk of misdiagnosing MM as MGUS. In our population-based registry, 5.5% of MM patients had MGUS-like MM. Although bone marrow sampling did not have therapeutic implications in our population, our findings indicate that even in patients referred with low M-protein concentrations, normal sFLC ratio and normal levels of hemoglobin, creatinine and ionized calcium, careful evaluation of symptoms and sensitive imaging techniques should be considered.

## Supplementary information

Revised supplementary material
