# Supplementary material for: Incidence and clinical characteristics of multiple myeloma with low M-protein levels and normal values of hemoglobin, creatinine, calcium, and serum free light chain ratio
Source: Blood Cancer J. 2021 Apr 7;11(4):70. doi: 10.1038/s41408-021-00460-0 (PMC8027460; doi:10.1038/s41408-021-00460-0)
Supplement: Supplementary file 1 — Revised supplementary material [file 41408_2021_460_MOESM1_ESM.docx]

Supplementary material

**Incidence and clinical characteristics of multiple myeloma with low M-protein levels and normal values of hemoglobin, creatinine, calcium and serum free light chain ratio**

Agoston Gyula Szabo^1^, Tobias Wirenfeldt Klausen^2^, Niels Abildgaard^3^ Henrik Gregersen^4^, Trine Silkjær^5^, Per Trøllund Pedersen^6^, Robert Schou Pedersen^7^, Carsten Helleberg^2^, Emil Hermansen^8^, Brian Iversen^1^, Annette Juul Vangsted^9^

1: Department of Hematology Vejle Hospital, Vejle, Denmark

2: Department of Hematology Herlev University Hospital, Herlev, Denmark

3: Department of Hematology Odense University Hospital, Odense, Denmark

4: Department of Hematology Aalborg University Hospital, Aalborg, Denmark

5: Department of Hematology Aarhus University Hospital, Aarhus, Denmark

6: Department of Hematology Esbjerg Hospital, Esbjerg, Denmark

7: Department of Hematology Regionshospitalet Holstebro, Holstebro, Denmark

8: Department of Hematology Zealand University Hospital, Roskilde, Denmark

9: Department of Hematology Rigshospitalet, Copenhagen, Denmark

Corresponding author:

Agoston Gyula Szabo, MD, Senior Registrar

Vejle Hospital

Department of Hematology Beriderbakken 4, 7100 Vejle

Tel no: +45 24784962

e-mail: agoston.gyula.szabo@rsyd.dk

Supplementary Figure 1

Correlation between the incidence of multiple myeloma and the proportion of patients assessed with sensitive imaging techniques


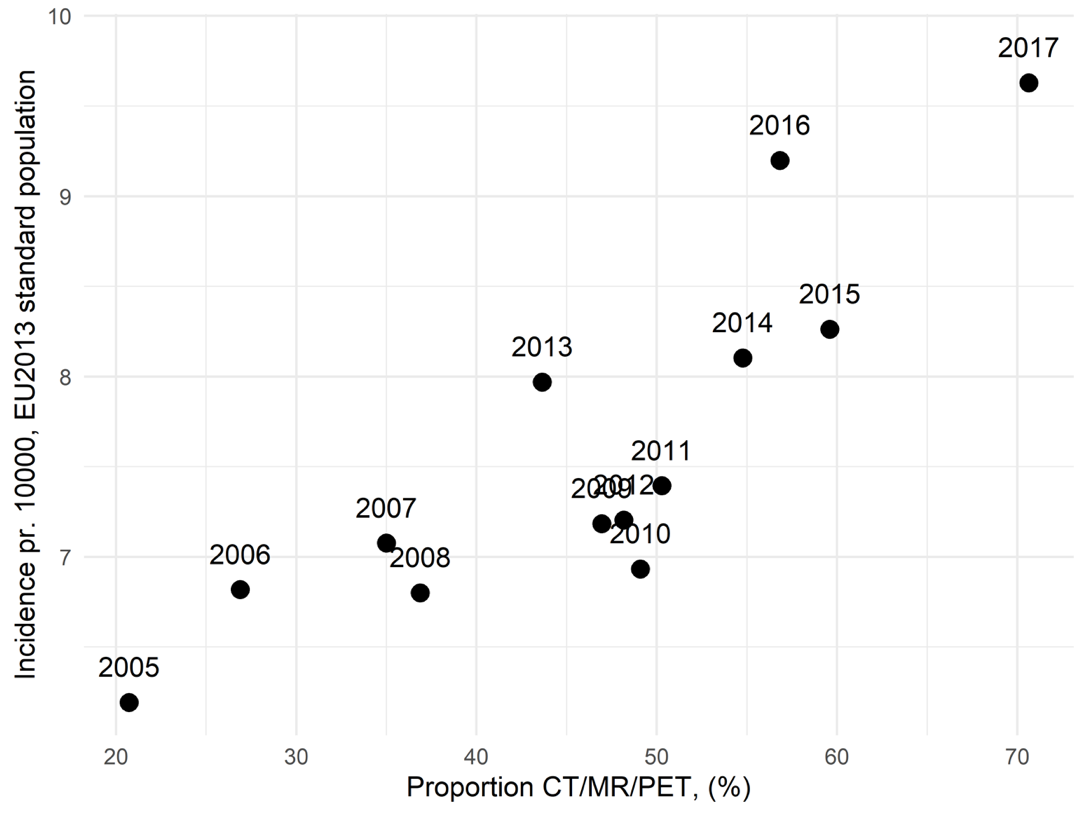


The vertical axis shows the incidence of multiple myeloma pr. 10.000 in Denmark, age adjusted to the EU2013 standard population. The horizontal axis shows the proportion of patients (%) assessed with sensitive imaging techniques like CT, MRI and PET (CT/MR/PET) as part of the diagnostic work-up. There was a significant correlation between the two variables (r=0.86; 95%CI: 0.58-0.96; p=0.0002). The years of diagnosis are shown separately in the figure, next to the black dots. Patients diagnosed in 2018 were not fully registered at data cut-off.

Supplementary Figure 2

Correlation between bone marrow clonal plasma cell infiltration and serum free light chain ratio


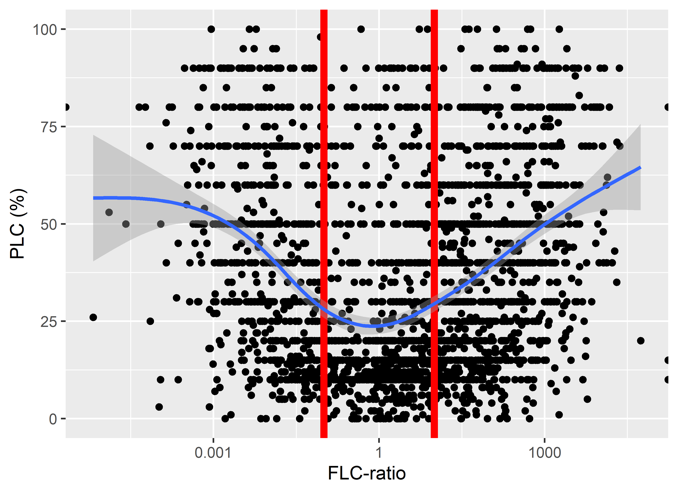


The vertical axis shows the percentage of bone marrow clonal plasma cell infiltration (PLC) in relation to serum free light chain (FLC) ratio presented on the horizontal axis. The red line illustrates the normal range of the serum free light chain ratio.

Supplementary Figure 3

Correlation between M-protein concentrations and bone marrow clonal plasma cell infiltration


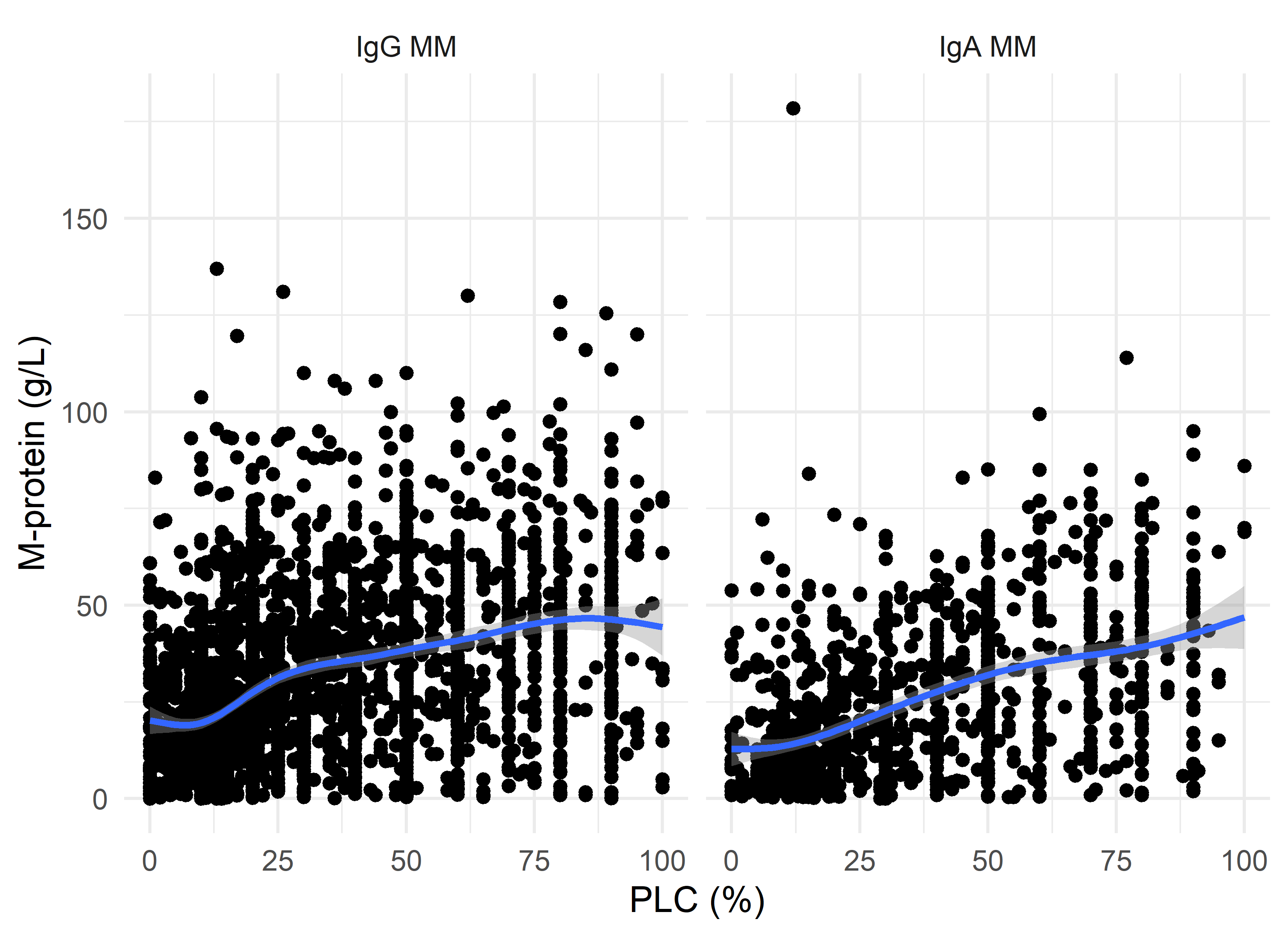


The vertical axis shows the levels of M-protein concentrations and the horizontal axis shows the percentage of bone marrow clonal plasma cell infiltration (PLC). Left panel: M-protein IgG (g/L). Right panel: M-protein IgA (g/L).

Supplementary Table 1

Characteristics of patients with IgG M-protein ≤1.5 g/dL, IgA M-protein ≤1.0 g/dL, normal blood levels of ionized calcium, creatinine, hemoglobin and normal serum free light chain ratio

|  |  | IgG M-protein ≤1.5 mg/dL | IgA M-protein ≤1.0 mg/dL | Total | Missing |
| --- | --- | --- | --- | --- | --- |
| N |  | 87 | 52 | 139 |  |
| Sex (F/M) | N | 53/34 | 35/17 | 88/ 51 |  |
| Age | Median (range) | 66 (32-86) | 68 (33-89) | 66 (32-89) |  |
| Bone marrow clonal plasma cells | Median (range) | 12 (0-50) | 12 (2-50) | 12 (0-50) | 2 |
| BMPC > 60% | N (%) | 0 (0) | 0 (0) | 0 | 2 |
| Osteolytic lesions | N (%) | 24 (27.9) | 9 (17.3) | 33 (23.9) | 1 |
| CT, PET or MRI done | N (%) | 55 (63.2) | 31 (59.6) | 86 (61.9) |  |
| Hemoglobin (mmol/L) | Median (range) | 8.5 (7.3-10.5) | 8.7 (7.4-10.0) | 8.6 (7.3-10.5) |  |
| Creatinine (μmol/L) | Median (range) | 71 (42-104) | 68 (34-102) | 70 (34-104) |  |
| Ionized calcium (mmol/L) | Median (range) | 1.24 (1.15-1.32) | 1.26 (1.10-1.32) | 1.25 (1.10-1.32) |  |
| Beta-2-microglobulin (mg/L) | Median (range) | 2.0 (1.2-4.7) | 2.1 (1.-5.9) | 2.0 (1.0-5.9) | 23 |
| Elevated Beta-2-microglobulin | N (%) | 24 (31.6) | 16 (40.0) | 40 (34.5) | 23 |
| Albumin (g/L) | Median (range) | 4.2 (2.9-5.0) | 4.2 (3.4-5.0) | 4.2 (2.9-5.0) | 3 |
| Low albumin | N (%) | 2 (2.3) | 1 (2.0) | 3 (2.2) | 3 |
| CRP (mg/L) | Median (range) | 3.0 (0.4-133) | 2.6 (0.2-29) | 2.9 (0.2-133) |  |
| Elevated CRP | N (%) | 16 (18.4) | 3 (5.8) | 19 (13.7) |  |
| LDH (units/L) | Median (range) | 184 (77-359) | 183 (89-267) | 184 (77-359) | 3 |
| Elevated LDH | N (%) | 15 (17.4) | 6 (12) | 21 (15.4) | 3 |
| Hypogammaglobulinemia | N (%) | 43 (49.4) | 30 (58.9) | 73 (52.9) | 1 |
| ECOG PS 0 | N (%) | 67 (77.0) | 31 (60.8) | 98 (71.0) | 1 |
| ECOG PS 1 | N (%) | 17 (19.5) | 19 (37.3) | 36 (26.1) |  |
| ECOG PS 2 | N (%) | 2 (2.3) | 1 (2.0) | 3 (2.2) |  |
| ECOG PS 3 | N (%) | 1 (1.1) | 0 (0) | 1 (0.7) |  |
| ECOG PS 4 | N (%) | 0 (0) | 0 (0) | 0 (0) |  |

Characteristics of MM patients with IgG≤ 1.5g/dl, IgA≤ 1.0g/dl and with normal levels of ionized calcium, creatinine, hemoglobin, serum free light chain ratio and no amyloidosis, medullary compression syndrome, extramedullary myeloma, peripheral neuropathy or dialysis-dependent renal failure at presentation.

Supplementary Table 2

Causes of diagnostic work-up of myeloma patients with IgG M-protein ≤1.5 g/dL, IgA M-protein ≤1.0 g/dL, normal blood levels of ionized calcium, creatinine, hemoglobin and normal serum free light chain ratio

| Causes of diagnostic work-up: N. (%) | Further specification of cause: N. (% of subgroup) |
| --- | --- |
| Symptoms: 80 (58.0) | Bone pain: 37 (46.3)  Fatigue: 12 (15.0)  Infections: 7 (8.8)  Constitutional symptoms: 9 (11.3)  Other: 45 (56.3) |
| Blood and urine tests: 91 (65.9) | M-protein in serum 87 (95.6)  M-protein in urine 1 (1.1)  Abnormal serum free light chain ratio at the time referral but not at diagnosis: 9 (9.9)  Proteinuria: 2 (2.2)  Other: 15 (16.5%) |
| Abnormal skeletal imaging results: 14 (10.1) | X-ray of skeleton: 8 (57.1)  CT: 5 (35.7)  MRI: 5 (35.7)  Other: 1 (7.1) |
| Biopsy: 8 (5.8) | Bone marrow biopsy: 1 (12.5)  Biopsy of bone-related lesion: 7 (87.5) |

Causes of diagnostic work-up were collected by audit of the medical records. Data were available in 138 patients (medical records in one patient were inaccessible). Several causes of diagnostic work-up are displayed per patient.
